# Assessment of Occupational Exposure to Blood and Other Body Fluids Among Healthcare Workers in a South‐Western Chinese Tertiary Hospital From 2018 to 2023: A Descriptive Cross‐Sectional Study

**DOI:** 10.1002/nop2.70432

**Published:** 2026-01-05

**Authors:** Lulin Chen, Wei Liu, Chunting Dong, Jun Yang, Yongjiang Gan, Yao Zhong, Danyan Liang

**Affiliations:** ^1^ The Second Nanning People's Hospital Nanning People's Republic of China; ^2^ Guangxi University of Science and Technology Nanning People's Republic of China

**Keywords:** healthcare worker, incidence rate, needlestick and sharps injuries, occupational exposure

## Abstract

**Aim:**

Healthcare workers face significant occupational exposure risks from biological and non‐biological hazards, with needlestick injuries being the most common hazard. This study aimed to assess the extent of occupational exposure among various healthcare workers.

**Design:**

A descriptive cross‐sectional study.

**Methods:**

This study was conducted from 2018 to 2023 in a south‐western Chinese tertiary hospital. The data about occupational exposure to blood and other body fluids were collected, including characteristics of healthcare workers and source patients, characteristics of such exposure and circumstances leading to such exposure.

**Results:**

There were 675 occupational exposure events from 2018 to 2023, predominantly involving female (78.81%) and individuals under 30 years old (62.81%). All of the individuals had received training (100.00%) and 58.52% had received vaccination against hepatitis B. The most common occupational exposure to blood and other body fluids was needlestick and sharps injuries (83.56%), with HBV being the primary disease associated with the exposure (22.96%). The incidence rate peaked in 2019 (60.38 per 1000 person‐years) and declined thereafter, with medical students, cleaning staffs and nurses experiencing the highest rate of exposure. The majority of incidents occurred in hospital wards (50.07%) and operating rooms (18.52%), often involving fingers (76.30%), and happened during patient care (17.63%), needle withdrawal (17.19%), and surgery operation (14.37%). Despite the high number of exposures, no medical staff contracted diseases from such exposure over the 6‐year period, as confirmed by a 6‐month serological follow‐up.

**Public Contribution:**

The highest incidence rate was observed in 2019, followed by a decline. Medical students, cleaning staffs and nurses had the highest occupational exposure incidence rates. The majority of the exposures occurred in hospital wards and operating rooms, primarily on the fingers and were most commonly caused by syringe needles or scalp needles. These results highlight the need for continued vigilance and targeted interventions to reduce the risk of occupational exposure among these groups, particularly in locations where such exposures are most common.

## 1 Introduction

Occupational exposure refers to the presence of a substance or risk factor in the work environment external to the worker (World Health Organization 2021a). Healthcare workers are at greater risk of occupational exposure. Among these risks, occupational exposure to bloodborne pathogens is the most common occupational hazard (Shi et al. 2020; Bouya et al. 2020; Tawiah et al. 2022). Blood‐borne pathogens encompass a wide spectrum of infectious agents that can be transmitted through contact with blood or other bodily fluids, posing a significant threat to the health and safety of healthcare workers, such as human immunodeficiency virus (HIV), hepatitis B virus (HBV) and syphilis. Protecting healthcare workers from harm caused by such pathogens has garnered more attention in terms of interventions such as strengthening adherence to standard precautions and systematic use of personal protective equipment (PPE) (McDiarmid 2014).

Occupational exposures to blood and other body fluids in the healthcare sector pose a threat to both low‐, middle‐ and high‐income countries. In high‐income countries, the implementation of comprehensive safety measures has significantly reduced the incidence of exposure to blood and other body fluids, but the lack of resources and prioritisation of occupational health has led to higher risks of exposure among healthcare workers in many low‐ and middle‐income countries (Macaia and Lapão 2017; Lucchini and London 2014). This disparity underscores the need for improved occupational health policies and practices in low‐ and middle‐income countries to better protect healthcare workers from such hazards. In addition to inadequate data collection systems, ineffective enforcement of health regulations, and the neglect of occupational health standards and guidelines are key factors contributing to deficiencies in occupational health work in some countries. Furthermore, the failure of healthcare workers to strictly adhere to standard precautions poses a serious concern (Tadesse and Dolamo 2022; Nuwayhid 2004; Persaud et al. 2023).

China is a country with a high prevalence of hepatitis B and faces a rapidly growing HIV epidemic (Wang and Cui 2022). This poses challenges for healthcare workers in terms of occupational safety. Previous studies have investigated occupational exposures to blood and other body fluids in China and other countries, suggesting that nurses are one of the primary subjects of such exposure around the world (Shi et al. 2020; Wang et al. 2023; Lee et al. 2017). Several studies also demonstrated that nursing students may be particularly susceptible to such exposure due to their insufficient technical skills, limited clinical experience and unfamiliarity with the working environment (Zhao et al. 2023). Therefore, occupational exposure to blood and other body fluids among various categories of healthcare workers may not be comprehensive enough. To contribute to the existing body of knowledge on this topic, we conducted this study to investigate the epidemiological features of such exposure in a hospital setting, which can provide insights to the global community, helping to develop more effective prevention strategies to reduce occupational exposure risks for nursing staff and enhance the overall safety of nursing workplaces.

## 2 Methods

### 2.1 Study Design and Period

**FIGURE 1:**
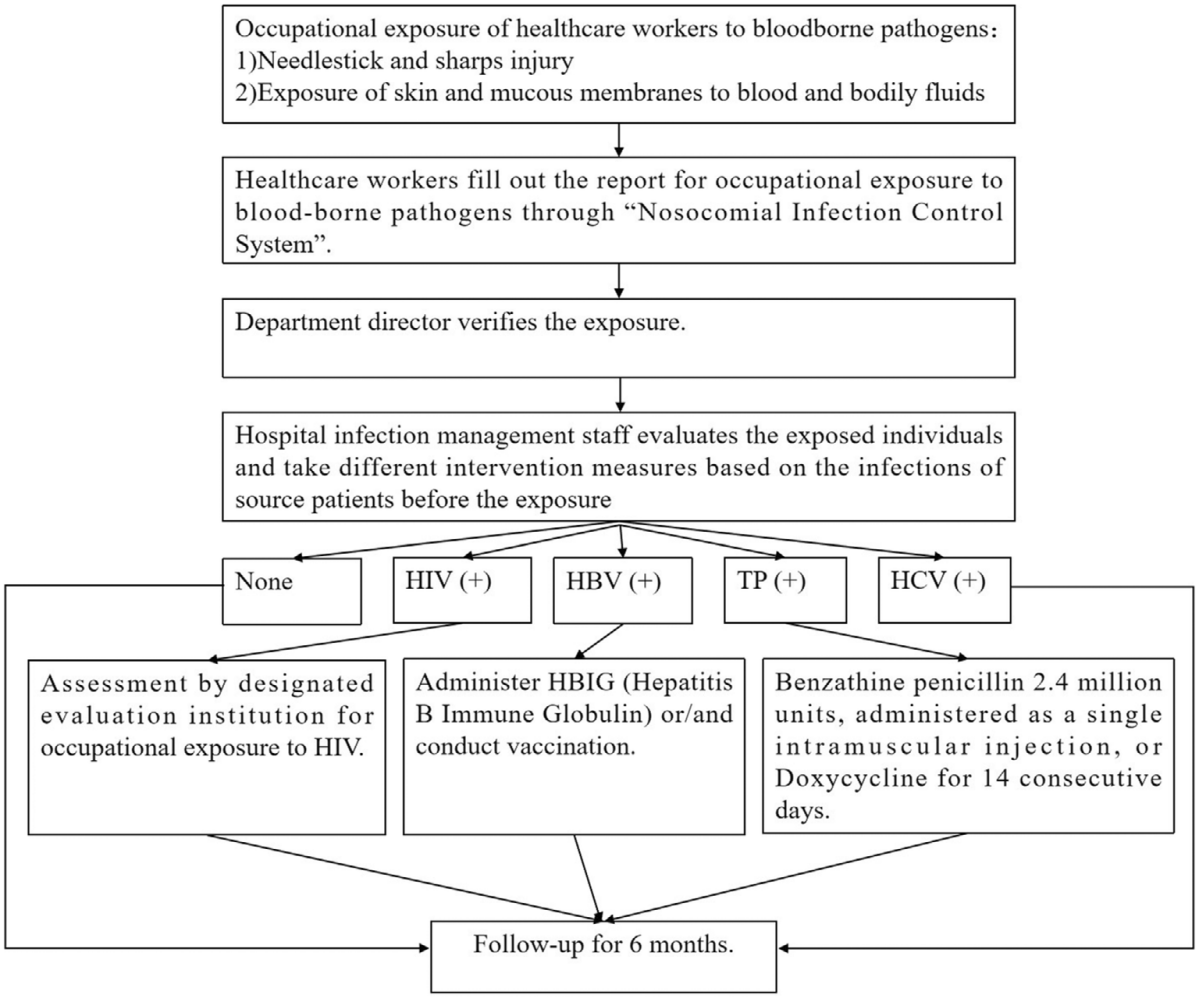
The study design framework.

This descriptive cross‐sectional study was conducted in a tertiary teaching hospital in south‐western China from January 2018 to December 2023. The reporting of this study followed the Strengthening the Reporting of Observational Studies in Epidemiology (STROBE) guideline. According to relevant regulations, healthcare workers are required to report occupational exposure to blood and other body fluids to the department of hospital infection control immediately in China. After being reviewed and confirmed by the department director, the report was submitted to our Hospital Infection Control Department through ‘Nosocomial Infection Control System’. Our Hospital Infection Control Department staff evaluated the exposed individuals, and different post‐exposure prophylaxes were provided according to relevant guidelines and regulations (Bijie et al. 2012). The ‘Nosocomial Infection Control System’ possesses the functionality to export reports, from which we derived data for secondary analysis. This strict documentation process ensures that all events are accurately recorded, which helps to improve reliability and data integrity. The study design is presented in Figure 1.

### 2.2 Study Setting

The research hospital is a comprehensive medical facility with an average of 2819 health workers per year and a bed capacity of 1160. It possesses comprehensive medical facilities, a professional medical team, and extensive clinical experience. The hospital has 46 clinical departments covering various fields such as internal medicine, surgery, obstetrics and gynaecology, paediatrics, enabling it to provide high‐quality medical services. Furthermore, the hospital boasts a specialised hospital infection control department with a staff of eight individuals, which possesses a rigorous quality control system and is responsible for monitoring and reporting occupational exposure incidents.

### 2.3 Data Sources and Measurements

The report included characteristics of healthcare workers, the infections of the source patients before the exposure, characteristics of occupational exposure to blood and other body fluids, and circumstances leading to occupational exposure to blood and other body fluids. All data used for analysis were directly extracted from the report, which was exported by the hospital's infection management staff from the ‘Nosocomial Infection Control System’ for further processing. HIV infection was confirmed by positive Elisa and western blotting. HBV infection was diagnosed by a positive HBsAg, HBeAg, HBcAb or detectable HBV DNA. HCV infection was diagnosed by HCV seropositivity or detectable HCV RNA. Positive results for both RPR and TPPA were considered to have a current syphilis infection. For inpatients, these screenings were performed prior to hospitalisation. For exposures occurring in the outpatient setting, screenings were performed within 2 h of exposure.

### 2.4 Inclusion and Exclusion Criteria

#### 2.4.1 Inclusion Criteria

All healthcare workers employed at the research hospital during the study period, regardless of occupation (doctors, nurses, medical students [including nursing students], residents, laboratory technicians, cleaning staff) who had an occupational exposure incident involving blood or other body fluids that was reported to the ‘Nosocomial Infection Control System’.

#### 2.4.2 Inclusion Criteria

Reports were excluded if the exposure route or body fluid type were missing, or irreconcilable data contradictions existed.

### 2.5 Sample Size Calculation

The sample size for this cross‐sectional study on occupational exposure to blood and other body fluids among healthcare workers in the hospital setting was determined based on the desired level of precision, estimated prevalence and confidence level. The study aimed to estimate the prevalence of occupational exposure to blood and other body fluids with a 95% confidence level and a desired margin of error of ±5%. Using available literature and preliminary data, the estimated prevalence was approximately 15%. Considering a *Z*‐score of 1.96 for a 95% confidence level, the formula for calculating the required sample size (*n*) was as follows: *n* = *Z* ^2^ × *p* × (1 − *p*)/*E* ^2^; where: *Z* = 1.96 (for a 95% confidence level), *p* = 0.15 (estimated prevalence of occupational exposure to blood and other body fluids as 15%), and *E* = 0.05 (desired level of precision as ±5%). To achieve the desired level of precision, a minimum sample size of 196 participants was calculated.

### 2.6 Ethical Approval

The current study was granted by the Ethics Review Committee (ERC) REDACTED. Given that the study exclusively utilised existing medical records and posed minimal risk to participants and did not involve the collection of new data from them, an application for a waiver of informed consent for the research was submitted to the ERC, which was subsequently granted.

### 2.7 Statistical Analyses

We conducted a descriptive analysis of characteristics of healthcare workers and source patients, occupational exposure rate and risk according to professions and years, characteristics of occupational exposure to blood and other body fluids, and circumstances leading to occupational exposure to blood and other body fluids. The incidence rate of occupational exposure was calculated per 1000 person years. The total number of healthcare workers each year was obtained from the hospital's human resources and education management departments, and grouped by categories. The study utilised SPSS 25.0 for analysis and Graphpad Prism 9.5 for graphical representation.

## 3 Results

During the 6 years (2018–2023), there was an average of 2819 health workers per year, with a total of 675 events of occupational exposure. The majority of these events involved women (532, 78.81%) and individuals under 30 years old (424, 62.81%). All of the exposed individuals had received training. Most of them had been vaccinated for hepatitis B (395, 58.52%). The majority of the occupational exposure to blood and other body fluids was needlestick and sharps injuries (564, 83.56%). The two most common diseases suffered by patients associated with the exposure were HBV (155, 22.96%) and syphilis (93, 13.78%) (Table 1).

**TABLE 1:** Characteristics of healthcare workers and source patients from 2018 to 2023 (*n* = 675).

Variables	*N* (%)
Healthcare workers characteristics	
Gender	
Male	143 (21.19)
Female	532 (78.81)
Age	
< 30	424 (62.81)
30–39	143 (21.19)
40–49	52 (7.70)
≥ 50	56 (8.30)
Hepatitis B vaccination	
Yes	395 (58.52)
No	232 (34.37)
Unknown	48 (7.11)
Training on occupational exposure operations	
Yes	675 (100.00)
No	0 (0.00)
Usage of PPE	
Yes	498 (73.78)
No	177 (26.22)
Mechanism of occurrence	
Needlestick and sharps injury	564 (83.56)
Percutaneous injury	111 (16.44)
Splash exposure	0 (0.00)
The infection of patients before the exposure	
HIV	
Positive	22 (3.26)
Negative	535 (79.26)
Unknown	118 (17.48)
HBV	
Positive	155 (22.96)
Negative	402 (59.56)
Unknown	118 (17.48)
HCV	
Positive	15 (2.22)
Negative	542 (80.30)
Unknown	118 (17.48)
Syphilis	
Positive	93 (13.78)
Negative	464 (68.74)
Unknown	118 (17.48)

**FIGURE 2:**
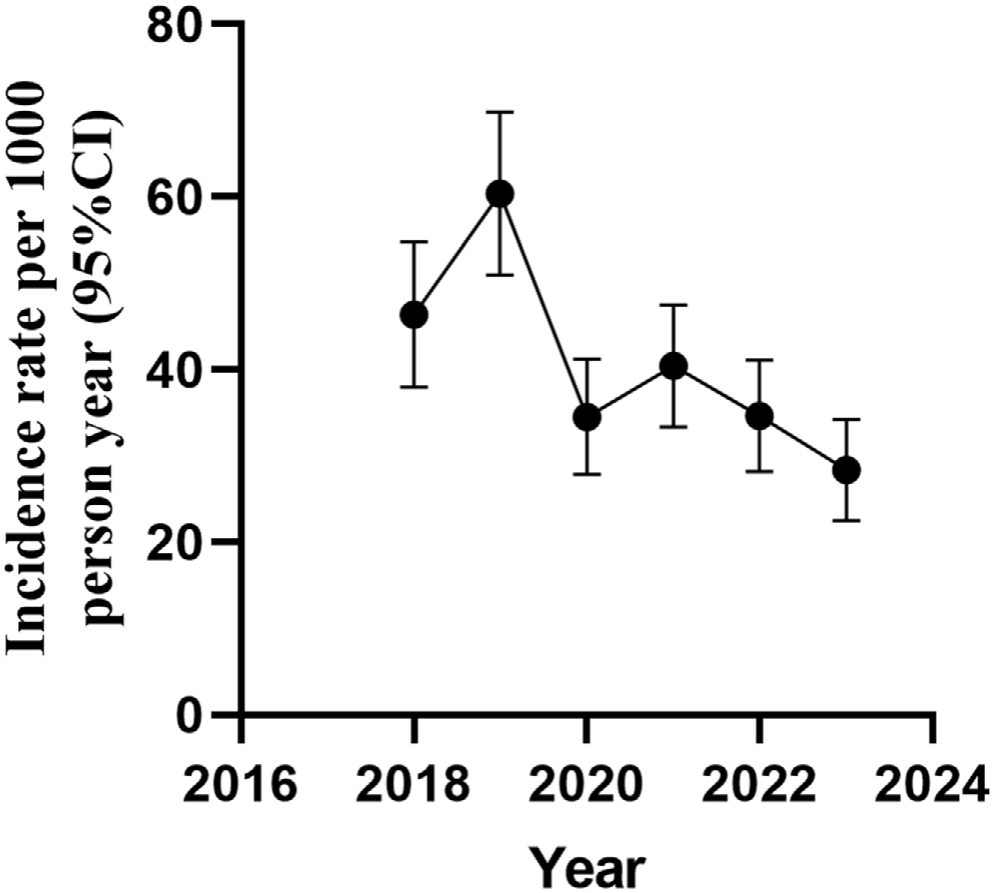
Incidence rate of occupational exposure according to years (per 1000 person‐year).

The average annual incidence rate of occupational exposure to blood and other body fluids over the 6 years was 39.90 per 1000 person‐years, peaking in 2019 at 60.38 per 1000 person‐years, followed by a decline (Figure 2). Among all reported events, the majority were nurses (35.26%), followed by medical students (30.22%) and doctors (17.48%). The incidence rate among medical students was the highest (64.97 per 1000 person‐years), followed by cleaning staffs (50.73 per 1000 person‐years) and nurses (42.37 per 1000 person‐years), significantly higher than that of other professions (Table 2).

**TABLE 2:** Incidence rate of occupational exposure to blood and other body fluids according to professions from 2018 to 2023 (*n* = 675).

Profession	Total number of persons at risk, *N*	Number of occupational exposures recorded, *N* (%)	Incidence per 1000 person‐years	95% CI
Nurses	5617	238 (35.26)	42.37	37.10–47.64
Medical students	3140	204 (30.22)	64.97	56.34–73.59
Medical doctors	3910	118 (17.48)	30.18	24.81–35.54
Medical residents	1805	48 (7.11)	26.59	19.16–34.02
Cleaning staffs	966	49 (7.26)	50.73	36.86–64.59
Laboratory technicians	1478	18 (2.67)	12.18	6.58–17.78

Most of the exposures occurred in hospital wards (50.07%) and operating rooms (18.52%), followed by ambulatory clinics/emergency rooms (13.48%). The majority of the exposures occurred on the fingers (76.30%), with syringe needles (30.67%) or scalp needles (26.52%) being the primary sources of these incidents (Table 3).

**TABLE 3:** Characteristics of occupational exposure to blood and other body fluids among healthcare workers from 2018 to 2023 (*n* = 675).

Variables	*N* (%)
Exposed place	
Ward	338 (50.07)
Operating room	125 (18.52)
Ambulatory clinic/emergency room	91 (13.48)
Nurses' station	54 (8.00)
ICU	31 (4.59)
Laboratory	18 (2.67)
Others	18 (2.67)
Exposed areas	
Finger	515 (76.30)
Conjunctiva of the eye	75 (11.11)
Palm of the hand	33 (4.89)
Back of the hand	32 (4.74)
Others	20 (2.96)
Devices related to occupational exposure	
Syringe needle	207 (30.67)
Scalp needle	179 (26.52)
Suture needle	66 (9.78)
Puncture needle	40 (5.93)
Acupuncture needle	25 (3.70)
Scalpel	28 (4.15)
Blood and bodily fluids	111 (16.44)
Others	19 (2.81)

17.63% of the exposures occurred while treating or caring for patients, 17.19% while withdrawing needles and 14.37% during surgical operations (Table 4). After the exposure occurred, a 6‐month serological follow‐up was conducted for the exposed individuals, and the monitoring results showed that there were no healthcare workers contracting diseases due to the exposure over the past 6 years.

**TABLE 4:** Circumstances leading to occupational exposure to blood and other body fluids (*n* = 675).

Variables	*N* (%)
Treating or caring for a patient	119 (17.63)
Needle withdrawal	116 (17.19)
Surgical operation	97 (14.37)
Arteriovenous puncture or percutaneous injection	83 (12.30)
Suffering a puncture wound from improperly placed sharp instruments	72 (10.67)
Disposing of used sharp instruments	57 (8.44)
Discarding needles and other sharp instruments	56 (8.30)
Capping a needle with both hands	49 (7.26)
Handling specimens	13 (1.93)
Others	13 (1.93)

## 4 Discussion

During the 6‐year period, healthcare workers reported 675 events of occupational exposure to blood and other body fluids, predominantly among women and individuals under 30 years old. All exposed individuals had undergone training, and most were vaccinated for hepatitis B. The most common exposures were needlestick and sharps injuries, with HBV and syphilis being the major sources of infection among patients. The incidence rate peaked in 2019 at 60.38 per 1000 person‐years, with medical students (including nursing students), cleaning staffs and nurses demonstrating the highest incidence rate. Exposures mostly occurred in hospital wards and operating rooms, primarily affecting the fingers and involving syringe or scalp needles. The most common circumstances leading to occupational exposure to blood and other body fluids were during patient treatment or care, needle withdrawal and surgical operations.

All of the exposed individuals had received training, but the majority involved women (532, 78.81%) and individuals under 30 years old (424, 62.81%). The predominance of women in the reported events may be attributed to the demographics of the hospital workforce, where women are often overrepresented in nursing (Mengistu and Tolera 2020). This could be a factor in the higher number of events among women. Younger healthcare workers might be more susceptible to occupational exposure due to limited experience and potentially less awareness of workplace hazards (Debelu et al. 2023). Despite all individuals having received training, the occurrence of these cases suggests that there might be a gap in the training programs' effectiveness. It could be that the training is not adequately targeted to the specific risks faced by these demographics or that the training does not translate into standard behaviours in the workplace (Qianqian et al. 2022). Our result verified that needlestick and sharps injuries are the most frequently experienced occupational exposure to blood and other body fluids among healthcare workers (Mengistu and Tolera 2020; Hosseinipalangi et al. 2022).

The average annual incidence rate of occupational exposure over the 6 years was 39.90 per 1000 person‐years, peaking in 2019 at 60.38 per 1000 person‐years, higher than the incidence rate reported in other countries, with an incidence rate between 13.3 and 45.0 per 1000 person‐years (Lee et al. 2017; Yunihastuti et al. 2020; Seng et al. 2016; Garus‐Pakowska and Górajski 2019; Çiçek‐Şentürk et al. 2019; Ishak et al. 2019). While our data was derived from a voluntary reporting system, it is important to recognise the potential for underreporting of occupational exposure incidents. There is a prevalent tendency among healthcare workers to avoid reporting work‐related events. Studies have indicated that the rate of underreporting can range significantly, from 42% to as high as 95%. This suggests that the actual situation could be more concerning than the reported data. The significant decline in exposure rates after the peak of 2019 could be attributed to several factors related to the COVID‐19 pandemic: (1) Enhanced PPE usage: the pandemic has led to an increased and consistent use of PPE among medical staff, which is a primary preventive measure against occupational exposure to blood and other body fluids and airborne infections (Zhao et al. 2020; McKenna et al. 2024); (2) Improved infection control protocols: hospitals had to reinforce or introduce new infection control protocols to manage the spread of COVID‐19. These protocols often include more frequent hand hygiene, environmental cleaning and the safe handling of sharps, which can contribute to a decrease in occupational exposure incidents (World Health Organization 2023; Gozdzielewska et al. 2022); (3) Training and education: There has likely been a surge in training programs to educate healthcare workers on the risks of occupational exposure and the importance of following health guidelines, especially during the pandemic (Dedeilia et al. 2023; Nayahangan et al. 2021; Persaud and Mitchell 2021); (4) Healthcare worker surveillance and support: The pandemic has highlighted the need for the surveillance of healthcare workers' health and the provision of psychological support (World Health Organization 2020; Meredith et al. 2024); (5) Risk assessment and management: The necessity to assess and manage the risks associated with COVID‐19 may have led to more rigorous risk assessment practices being adopted for all occupational hazards, not just those related to the virus (Zhou et al. 2022). These pandemic‐driven interventions collectively reduced exposure incidence, with multiple studies corroborating these findings (Koscak et al. 2024; Jalilian et al. 2024).

The reported distribution of occupational exposures to blood and other body fluids among healthcare workers, with nurses, medical students (including nursing students) and doctors comprising the majority of events, provided a snapshot of the varying levels of risk these groups face in their work environments. The higher incidence rate among medical students (including nursing students), despite their numerical minority in the reported cases, suggests that this group may be at a particularly high risk. Nurses often have close and frequent contact with patients, which can increase their risk of exposure to blood and body fluids (Zhang et al. 2022). Medical students, especially during their clinical rotations, may have a higher risk of occupational exposure due to their relative inexperience and the learning curve associated with performing medical procedures. The higher incidence rate among medical students could be attributed to their involvement in a variety of procedures where there is a risk of exposure, such as drawing blood or assisting in surgical procedures. This is supported by studies that have reported on the prevalence of needlestick and sharps injuries and other forms of occupational exposure among medical students (Birenbaum et al. 2002). The incidence rate being highest among medical students suggests that there may be a need for more effective training on infection control and the use of universal precautions (Persaud et al. 2023; Mengistu et al. 2022). The findings highlight the critical need for targeted interventions to protect nursing staff and nursing students. Nurses, being the largest group of healthcare workers, are often at the forefront of patient care and thus face significant risks. The high incidence of exposure among nurses underscores the importance of continuous training and reinforcement of safety protocols. Additionally, the involvement of nursing students in the study highlights the need for robust educational programs that emphasise the prevention of occupational exposures. These programs should be integrated into nursing curricula to ensure that future nurses are well‐prepared to handle the risks associated with their profession.

The lower rate of occupational exposures among laboratory technicians may be due to several key reasons. Firstly, they work in well‐controlled lab environments with strict safety rules and always wear protective gear like gloves and goggles (Aldhamy et al. 2023). Secondly, their work is more routine and involves less direct contact with patients, reducing the risk of exposure to infections (Mangalea et al. 2024). Furthermore, lab technicians receive specialised training that focuses on safety and handling hazardous materials. Advanced technology, such as automated testing systems, helps reduce manual handling of samples and further lowers the risk. Lastly, the relatively lower stress levels in labs allow technicians to stay focused on safety protocols. All these factors together help keep the incidence of occupational exposures low among lab technicians.

It is common for the majority of occupational exposures to blood and other body fluids to occur in hospital wards and operating rooms, as these are areas with a high frequency of patient care and medical procedures that involve sharp instruments or contact with bodily fluids. Ambulatory clinics and emergency rooms would also be expected to have a significant number of exposures due to the urgent and varied nature of patient encounters. The nature of these settings often requires quick actions and a higher number of invasive procedures, which can increase the risk of occupational exposure (Gül 2021; Luo et al. 2021). Fingers, being the most common exposed body part, are consistent with the types of tasks performed by healthcare workers. Suturing, drawing blood, administering injections and other procedures frequently involve the hands and fingers, making them more susceptible to occupational exposures to blood and other body fluids and contact with infectious materials (Xu et al. 2021; Alfulayw et al. 2021). Syringe needles and scalp needles, being the most common sources of exposure, are also expected. These tools are frequently used in a variety of medical procedures and, due to their sharp nature, present a high risk of causing injury if not handled properly (NSW Health 2024; Sun et al. 2021).

The circumstances leading to occupational exposure were concerning as they highlight the critical moments during which healthcare workers are at risk. The fact that a significant percentage of exposures occur while treating or caring for patients underscores the need for rigorous infection prevention and control measures to be in place at all times in patient care settings (Zhang et al. 2022). This includes the use of appropriate PPE, adherence to hand hygiene protocols and safe handling of medical equipment (Persaud et al. 2023). The high incidence of exposure while withdrawing needles is a stark reminder of the risks associated with needlestick and sharps injuries (Zhang et al. 2022). This reinforces the WHO's call for the global use of safety‐engineered syringes (World Health Organization 2021b), which are designed to reduce the risk of needlestick and sharps injuries. Similarly, the occurrence of occupational exposures to blood and other body fluids during surgical operations indicates the need for enhanced infection prevention and control practices in the operating room. This could involve strict protocols for the handling of sharps, use of protective gear and immediate response measures in case of an accidental exposure. The fact that no medical staff contracted diseases due to occupational exposure over a 6‐year period was a positive outcome and suggests that the hospital's infection control and post‐exposure management protocols were effective (Labhardt et al. 2021).

Although the research provides valuable insights into occupational exposures among healthcare workers, there are several limitations worth noting. Firstly, the study covered an average of 2819 health workers per year, but the total number of cases (675) may not be representative of a broader population or specific regions. A larger sample size could provide more robust results. Secondly, the study relied on self‐reported cases of occupational exposure, which may be subject to underreporting or misreporting. Finally, some risk factors that were not mentioned, such as work environment, workflow and personal behaviours, were not fully explored. A more comprehensive analysis could provide a more holistic understanding of risk factors.

## 5 Conclusions

The highest incidence rate was observed in 2019, followed by a decline. Medical students, cleaning staffs, and nurses had the highest occupational exposure incidence rates. The majority of the exposures occurred in hospital wards and operating rooms, primarily on the fingers, and were most commonly caused by syringe needles or scalp needles. These results highlight the need for continued vigilance and targeted interventions to reduce the risk of occupational exposure among these groups, particularly in locations where such exposures are most common.

## Author Contributions

Lulin Chen analysed the data and wrote the main manuscript text. Wei Liu revised this manuscript. Danyan Liang designed this study. Chunting Dong, Jun Yang, Yongjiang Gan and Yao Zhong collected the data. All authors have read and approved the final manuscript.

## Funding

This work was supported by self‐funded research project of The Health Committee of Guangxi Zhuang Autonomous Regions, Grant/Award Number (Z20210500).

## Ethics Statement

The study was approved by the ERC of our institution (No. Y2024288).

## Consent

An application for a waiver of informed consent for the research was submitted to the ERC, which was subsequently granted.

## Conflicts of Interest

The authors declare no conflicts of interest.

## Data Availability

The data are available from the corresponding author on reasonable requests.
